# CircHIPK3/miR-381-3p axis modulates proliferation, migration, and glycolysis of lung cancer cells by regulating the AKT/mTOR signaling pathway

**DOI:** 10.1515/biol-2020-0070

**Published:** 2020-09-06

**Authors:** Feng Gu, Junhan Zhang, Lin Yan, Dong Li

**Affiliations:** Department of Aspiration Oncology, Gansu Provincial Tumor Hospital, Lanzhou, Gansu, China; Department of Research and Experimental Center, Gansu University of Chinese Medicine, Lanzhou, Gansu, China; Department of Anesthesiology, Gansu Provincial Hospital, Lanzhou, Gansu, China; Department of Thoracic Surgery, Gansu Provincial Tumor Hospital, No. 2 Xiaoxihu East Street, Qilihe District, Lanzhou, Gansu, China

**Keywords:** lung cancer, circHIPK3, miR-381-3p, glycolysis, AKT/mTOR signaling pathway

## Abstract

Lung cancer is a lethal malignancy. Plenty of circular RNAs (circRNAs) have been identified to be the vital regulators in lung cancer development. Here, we intended to clarify the functional role of circRNA HIPK3 (circHIPK3, also called hsa_circ_0021593) and its underlying mechanism of action. Quantitative reverse transcription-polymerase chain reaction (qRT-PCR) was employed to evaluate the levels of circHIPK3 and miR-381-3p. Cell viability and apoptosis rate were monitored by Cell Counting Kit-8 assay and flow cytometry, respectively. Cell migration was estimated through the Transwell assay. To assess glycolysis, commercial kits were utilized to measure the levels of glucose and lactate and the enzyme activity of hexokinase-2 (HK2). Expression of related proteins was detected via western blot analysis. The target connection between circHIPK3 and miR-381-3p was validated by dual-luciferase reporter, RIP, and pull-down assays. The role of circHIPK3 *in vivo* was determined via the xenograft assay. CircHIPK3 was upregulated, while miR-381-3p was downregulated in lung cancer tissues and cells. And circHIPK3 deficiency inhibited lung cancer progression by lowering cell proliferation, migration, glycolysis, and promoting apoptosis of lung cancer cells *in vitro*. MiR-381-3p was a target of circHIPK3, and miR-381-3p interference alleviated circHIPK3 knockdown-induced lung cancer progression inhibition. CircHIPK3 could activate the protein kinase B/mammalian target of rapamycin (AKT/mTOR) signaling pathway. Moreover, circHIPK3 knockdown suppressed tumor growth *in vivo* by inactivating the AKT/mTOR signaling pathway. In conclusion, the silencing of circHIPK3 inhibited lung cancer progression, at least in part, by sponging miR-381-3p and inactivating the AKT/mTOR signaling pathway.

## 1 Introduction

Lung cancer is the leading cause of cancer death worldwide, and people exposed to smoking are at a higher risk of lung cancer [1]. Targeted therapy of lung cancer based on genomic profiling is a novel and promising approach [2]. Hence, a better understanding of the molecular mechanism of lung cancer development might be beneficial for targeted therapy.

Emerged as star molecules in cancer, circular RNAs (circRNAs) are a category of endogenous non-coding RNAs without 5′-caps and 3′-poly(A) tails; this makes them stable and resistant to digestion by ribonucleases [3]. Dysregulated circRNAs have been manifested to be closely related to the proliferation and metastasis of non-small cell lung cancer (NSCLC) cells and have the potential to be diagnostic biomarkers of lung cancer [4,5]. For instance, circCMPK1 (hsa_circ_0012384) positively modulated cell proliferation of NSCLC cells by increasing cyclin D1 expression by sponging miR-302e [6]. Li et al. reported that circ_0000003 could accelerate the progression of NSCLC by promoting the proliferation and metastasis via the miR-338-3p/IRS2 pathway [7]. Gao and his colleagues alleged that hsa_circ_0007059 inhibited lung cancer cell growth and epithelial–mesenchymal transition through the inhibition of miR-378 [8]. Derived from homeodomain-interacting protein kinase 3 (HIPK3), circRNA HIPK3 (circHIPK3) (ID: hsa_circ_0021593 in circBase; Position: chr11:33279167-33369559) is a well-studied circRNA in human cancers, playing an oncogenic role in prostate cancer, nasopharyngeal carcinoma (NPC), and glioma [9,10,11], as well as in lung cancer [12]. The current study was designed to explore a novel mechanism of circHIPK3 in the progression of lung cancer.

There is evidence demonstrating that circRNAs are involved in competing endogenous RNA (ceRNA) network via sponging microRNAs (miRNAs) to regulate lung cancer development [13]. MiRNAs function in development, progression, and response to therapy of human diseases including lung cancer, serving as oncogenes or tumor suppressor genes [14,15]. MiR-381-3p exerted important roles in human oral squamous cell carcinoma [16], head-neck squamous cell carcinoma [17], and cervical cancer [18], playing a tumor suppressor role. Besides, miR-381-3p was reported to be associated with the circFGFR1 (hsa_circ_0084003)-mediated NSCLC progression [19]. Nevertheless, the role of miR-381-3p in circHIPK3-mediated cellular behaviors of lung cancer cells is still unknown. AKT/mTOR signaling pathway is a common signaling pathway, participating in the development of lung cancer [20,21].

In the current study, we analyzed the functional role of circHIPK3 in the proliferation, migration, and glycolysis of lung cancer cells, as well as in tumor growth. We also explored the possible mechanism behind it.

## 2 Materials and methods

### 2.1 Human tissue collection

Forty-five paired human lung cancer tissues and adjacent healthy tissues were obtained from the patients hospitalized at Gansu Provincial Tumor Hospital. These patients were graded based on the AJCC Cancer Staging Manual, 7th edition, containing 24 cases (stages I and II) and 21 cases (stages III and IV).

**Informed consent:** Informed consent has been obtained from all individuals included in this study.

**Ethical approval:** The research related to human use has been complied with all the relevant national regulations, institutional policies and in accordance with the tenets of the Helsinki Declaration, and has been approved by the Ethics Committee of Gansu Provincial Tumor Hospital.

### 2.2 Cell culture and transient transfection

Human lung cancer H1975 (ATCC^®^ CRL-5908; ATCC, Manassas, VA, USA), A549 (ATCC^®^ CCL-185), and H1299 (ATCC^®^ CRL-5803D) cells and human bronchial epithelial cell line (HBE1; ENC002; Kerafast, Boston, MA, USA) were cultured in Dulbecco’s Modified Eagle Medium (Gibco, Grand Island, NY, USA) supplemented with 10% fetal bovine serum (Gibco), 100 IU/mL penicillin (Gibco), and 100 μg/mL streptomycin (Gibco) at 37°C in an atmosphere of 5% CO_2_.

For circHIPK3 silencing, small hairpin RNA (shRNA) against circHIPK3 (sh-circHIPK3) and its control (sh-NC) were designed and synthesized by Genomeditech (Shanghai, China). For circHIPK3 overexpression, the overexpression vector of circHIPK3 (circHIPK3) and its control (Vector) were supplied by Hanbio Biotechnology Co., Ltd (Shanghai, China). Besides, miR-381-3p mimic (miR-381-3p), mimic control (miR-NC), miR-381-3p inhibitor (anti-miR), and inhibitor control (anti-NC) were constructed by GeneCopoeia (Guangzhou, China). The aforementioned oligonucleotides and plasmids were introduced into lung cancer cells using Lipofectamine 2000 reagent (Invitrogen, Carlsbad, CA, USA).

### 2.3 Quantitative reverse transcription-polymerase chain reaction (qRT-PCR)

For detecting circHIPK3 expression, total RNA derived from lung cancer tissues, nearby healthy tissues, lung cancer cells, or HBE1 cells was isolated using TRIzol reagent (TaKaRa, Dalian, China), and then reverse-transcribed into complementary DNA (cDNA) with miScript II RT Kit (Qiagen, Frankfurt, Germany) referring to the manufacturers’ guidelines. Then, qPCR was performed using QuantiTect SYBR Green PCR Kit (Qiagen). As for miR-381-3p, miRNA was isolated with miRNeasy Mini Kit (Qiagen), and then reverse transcription and qPCR were conducted with miScript Reverse Transcription Kit (Qiagen) and TaqMan miRNA assays (Applied Biosystems Inc., Foster City, CA, USA), respectively. All primers involved in the qPCR assay were as follows: circHIPK3: 5′-GGATTGCACATGTTGTCTGG-3′ (sense) and 5′-AGAGGAAACGGCGAAACAC-3′ (anti-sense); HIPK3 mRNA: 5′-CTACAGATCCGACCAGGAGTTC-3′ (sense) and 5′-TGTGAACCAGCCACACTCTCAG-3′ (anti-sense); glyceraldehyde-3-phosphate dehydrogenase (GAPDH): 5′-GTCAAGGCTGAGAACGGGAA-3′ (sense) and 5′-AAATGAGCCCCAGCCTTCTC-3′ (anti-sense); miR-381-3p: 5′-TACTTAAAGCGAGGTTGCCCTT-3′ (sense) and 5′-GGCAAGCTCTCTGTGAGTA-3′ (anti-sense); and U6: 5′-CTCGCTTCGGCAGCACATATACT-3′ (sense) and 5′-ACGCTTCACGAATTTGCGTGTC-3′ (anti-sense). The relative expression of circHIPK3 and miR-381-3p was calculated by the 2^−ΔΔCt^ method, with GAPDH (for normalizing circHIPK3 and HIPK3 mRNA) or U6 (for normalizing miR-381-3p) serving as an internal reference.

### 2.4 Detection of cell viability

The viability of lung cancer H1975 and A549 cells was determined using Cell Counting Kit-8 (CCK-8; Boster Bio, Wuhan, China) based on the manufacturer’s instructions. Transfected cells seeded in 96-well plates were cultured for 48 h, then 10 µL of CCK-8 reagent was dropped into each well. The absorbance of every well at 450 nm was recorded following 2 h incubation using a microplate reader (ARVOmx; PerkinElmer, Uberlingen, Germany).

### 2.5 Examination of cell apoptosis

After transfection for 48 h, the apoptotic rate of lung cancer cells was evaluated with Annexin V-fluorescein isothiocyanate (FITC) Apoptosis Detection Kit (Beyotime, Shanghai, China). In brief, cells were washed and resuspended in 200 µL of binding buffer containing 5 µL of Annexin V-FITC, then 10 µL of propidium iodide was added. After incubation for 15 min in the dark, apoptotic cells were detected using a flow cytometer (Merck KGaA, Darmstadt, Germany).

### 2.6 Transwell assay for cell migration

Cell migration was measured by Transwell assay with a chamber (8 µm in size; Millipore, Billerica, MA, USA). About 1 × 10^4^ transfected H1975 and A549 cells were plated into the top chambers containing medium without serum. The bottom ones were filled with medium with 10% fetal bovine serum. After 24 h, cells that had migrated to the base of bottom chambers were fixed using methanol, stained with crystal violet, observed under a microscope, and counted.

### 2.7 Glycolysis assessment

Hexokinase-2 (HK2) is a key enzyme of the first step in glycolysis [22]. Here, we measured the glucose consumption, lactate production, and HK2 enzyme activity to evaluate the glycolysis process in lung cancer cells. The levels of glucose (ab65333; Abcam, Shanghai, China) and lactate (ab83429; Abcam) as well as the enzyme activity of HK2 (ab136957; Abcam) were analyzed using the corresponding kits according to the recommended instructions.

### 2.8 Western blot

To prepare the protein samples for loading, transfected H1975 and A549 cells or tumor tissues were lysed using a radio-immunoprecipitation assay. After quantification, 40 µg of protein samples were subjected to separation through sodium dodecyl sulfate–polyacrylamide gel electrophoresis (SDS-PAGE) and transferred onto polyvinylidene difluoride (PVDF) membranes (Millipore). After blocking in non-fat milk, the membranes were incubated in Tris Buffered Saline Tween (TBST) containing primary antibody against Ki-67 (ab92742; Abcam), matrix metalloproteinase 9 (MMP-9; ab38898; Abcam), HK2 (ab209847; Abcam), phosphorylated protein kinase B (p-AKT; ab192623; Abcam), AKT (ab179463; Abcam), p-mammalian target of rapamycin (p-mTOR; ab109268; Abcam), mTOR (ab32028; Abcam), or GAPDH (ab181602; Abcam) and then incubated with secondary antibody (ab205718; Abcam). The blots were visualized using a chemiluminescence kit (Merck Millipore, Darmstadt, Germany). GAPDH served as a control.

### 2.9 Dual-luciferase reporter assay

The potential target miRNAs of circHIPK3 were predicted using the online database starBase v3.0 (http://starbase.sysu.edu.cn/). For the dual-luciferase reporter assay, the segmental sequence of circHIPK3 embracing the predicted binding sites of miR-381-3p was inserted into the pGL4 vector (Promega, Madison, WI, USA) to build wild-type luciferase reporter of circHIPK3 (wt-circHIPK3). Quick Change Site-Directed Mutagenesis kit (Agilent Technologies, Inc., Santa Clara, CA, USA) was used to mutate the binding sites, then mutant-type luciferase reporter of circHIPK3 (mut-circHIPK3) was compounded in a similar way. H1975 and A549 cells were introduced with wt-circHIPK3 or mut-circHIPK3 and miR-381-3p or miR-NC. After 48 h, relative luciferase activity was determined using the Dual-Luciferase reporter system (Beyotime) referring to the user’s manual.

### 2.10 RNA immunoprecipitation (RIP) assay

This assay was performed using an EZ-Magna RIP Kit (Millipore) referring to the manufacturer’s instructions. In brief, H1975 and A549 cells were harvested and lysed in RIP lysis buffer, and the generated cell lysate was incubated with magnetic beads that had been conjugated to anti-Ago2 antibody (ab32381; Abcam) or anti-IgG antibody (ab109761; Abcam), followed by rotation at 4°C overnight. Input and normal IgG were used as controls. Then, RNA in magnetic beads-binding complexes was isolated after Proteinase K digestion. Later, the enrichment of circHIPK3 and miR-381-3p was examined by the qRT-PCR assay.

### 2.11 Pull-down assay

The pull-down assay was also employed to validate the binding between circHIPK3 and miR-381-3p. About 50 nM of biotin-labeled miR-381-3p (bio-miR-381-3p) or its negative control (bio-miR-NC) synthesized by GenePharma Co. Ltd. (Shanghai, China) was introduced into H1975 and A549 cells. After 48 h, the cells were lysed in lysis buffer. The cell lysate was incubated with M-280 Streptavidin beads (Invitrogen) at 4°C for 3 h. The bound RNA was isolated using TRIzol reagent and subjected to the qRT-PCR assay for analyzing circHIPK3 and HIPK3 mRNA expression levels.

### 2.12 Xenograft assay

Lentiviral-based shRNA targeting circHIPK3 (sh-circHIPK3) and its negative control were purchased from GeneChem (Shanghai, China). To construct the xenograft model, 5-week-old BALB/c nude mice (male) purchased from Shanghai SLAC Laboratory Animal Co., Ltd. (Shanghai, China) were hypodermically injected with H1975 cells stably expressing sh-circHIPK3 or sh-NC. The volume of generated tumors was monitored every 5 days and calculated based on the following formula: volume = 0.5 × length × width^2^. At day 30 post injection, the generated tumors were excised, weighed, and used for qRT-PCR and western blot assays.

**Ethical approval:** The research related to animal use has been complied with all the relevant national regulations and institutional policies for the care and use of animals and has been approved by the Ethical Committee of Gansu Provincial Tumor Hospital.

### 2.13 Statistical analysis

All data in this study were generated from three independent experiments, and each group had three parallel biology repetitions. Statistical significance of these data was measured via Student’s *t*-test (for two groups) or one-way analysis of variance with Tukey’s *post-hoc* test (for three or more groups). Data were shown as mean ± standard deviation. When *P* < 0.05, it is considered to be significant.

## 3 Results

### 3.1 CircHIPK3 was highly expressed in lung cancer

**Figure 1:**
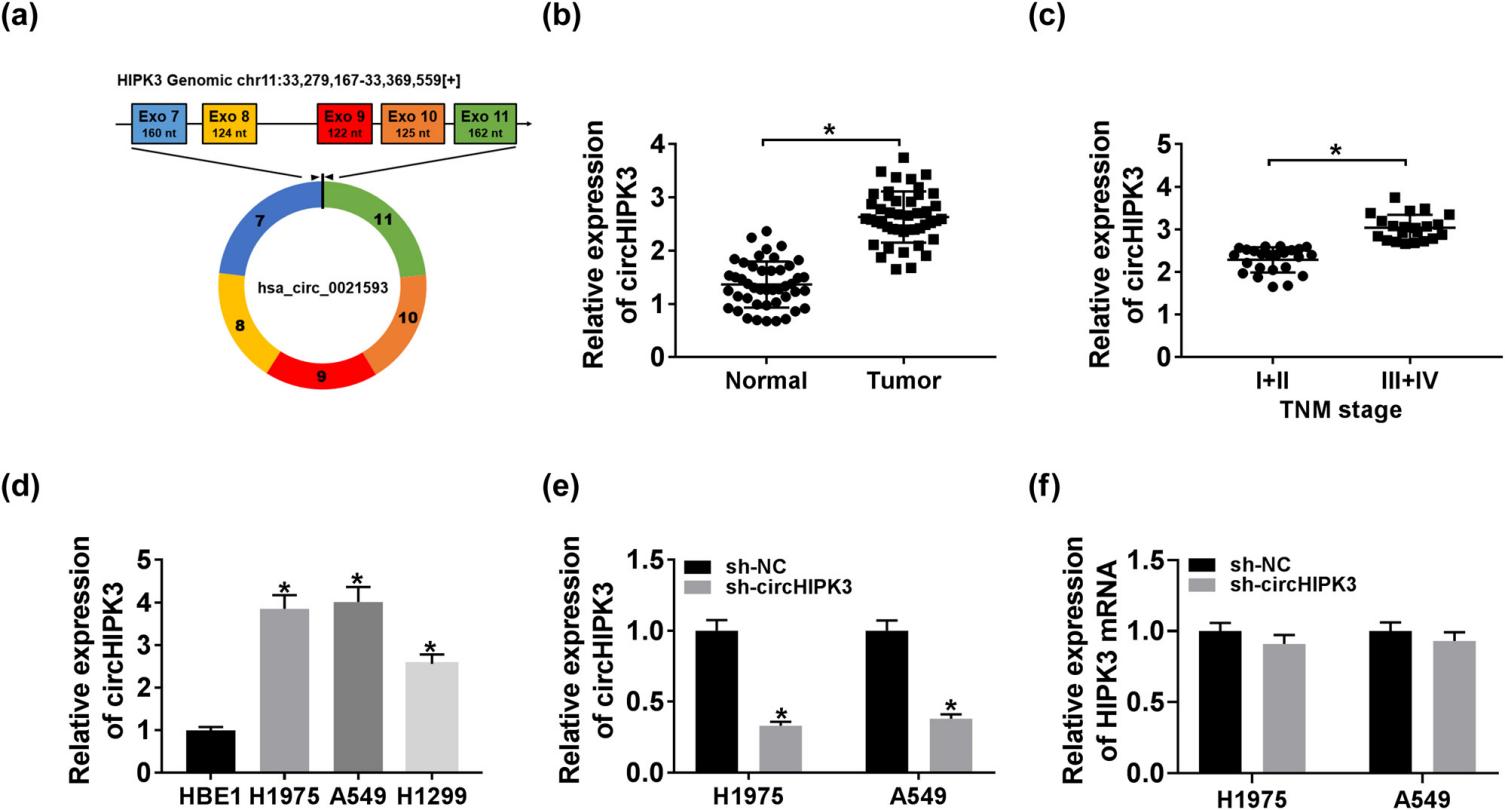
CircHIPK3 was highly expressed in lung cancer. (a) Schematic illustration showing HIPK3 exons 7–11 circularization to form circHIPK3 (black arrow). (b–d) QRT-PCR assay for the relative expression of circHIPK3 in lung cancer tissues and normal tissues (b), in lung cancer tissues at stages I and II and at stages III and IV (c), as well as in HBE1 cells and lung cancer H1975, A549, and H1299 cells (d). (e and f) QRT-PCR assay for the relative expression of circHIPK3 (e) and HIPK3 mRNA (f) in H1975 and A549 cells transfected with sh-NC or sh-circHIPK3. *P < 0.05.

Here, we first noted that circHIPK3 (chr11:33279167-33369559) is derived from exon 7, 8, 9, 10, and 11 regions within HIPK3 (Figure 1a), whose spliced mature sequence length is 2,576 bp. We then validated the ectopic upregulation of circHIPK3 in lung cancer tissues and cells. As illustrated in Figure 1b and c, circHIPK3 was enriched in lung cancer tissues (*n* = 45) compared to normal tissues, especially in lung cancer tissues at stages III and IV in contrast to tissues at stages I and II. To assess the significance of circHIPK3 in lung cancer, 45 patients were divided into a low circHIPK3 expression group (*n* = 22) and a high circHIPK3 expression group (*n* = 23) according to the median value of circHIPK3 expression in lung cancer tissues (Table 1). Then, the correlation between the expression level of circHIPK3 and clinical characteristics of lung cancer patients was analyzed. We found that the circHIPK3 expression was closely correlated with tumor size (*P* = 0.0003), TNM stage (*P* = 0.0003), and lymph node metastasis (*P* = 0.0009). Likewise, higher expression of circHIPK3 was detected in H1975, A549, and H1299 cells with respect to HBE1 cells (Figure 1d). To explore the effects of circHIPK3 on the cellular behaviors of lung cancer cells, H1975 and A549 cells were introduced with sh-circHIPK3 to knockdown circHIPK3, and sh-NC acted as the control. The knockdown efficiency of sh-circHIPK3 was determined by the qRT-PCR assay (Figure 1e). Meanwhile, we found that sh-circHIPK3 had no significant effect on the expression of HIPK3 mRNA (Figure 1f). In short, circHIPK3 was highly enriched in lung cancer.

**Table 1:** Correlation between circHIPK3 expression and clinical characteristic of patients with lung cancer (*n* = 45)

Variable	CircHIPK3 expression		*P* value
	Low (*n* = 22)	High (*n* = 23)	
Sex			
Male	5	8	0.513
Female	17	15	
Age (years)			
<50	8	12	0.373
≥50	14	11	
Tumor size (cm)			
<3	17	5	0.0003**
≥3	5	18	
TNM stage			
I–II	16	8	0.017*
III–IV	6	15	
Smoking history			
Never smokers	15	13	0.542
Smokers	7	10	
Lymph node metastasis			
No	16	5	0.0009**
Yes	6	18	

*P < 0.05, **P < 0.01.

### 3.2 CircHIPK3 knockdown repressed cell proliferation, migration, and glycolysis while facilitated apoptosis in lung cancer cells

**Figure 2:**
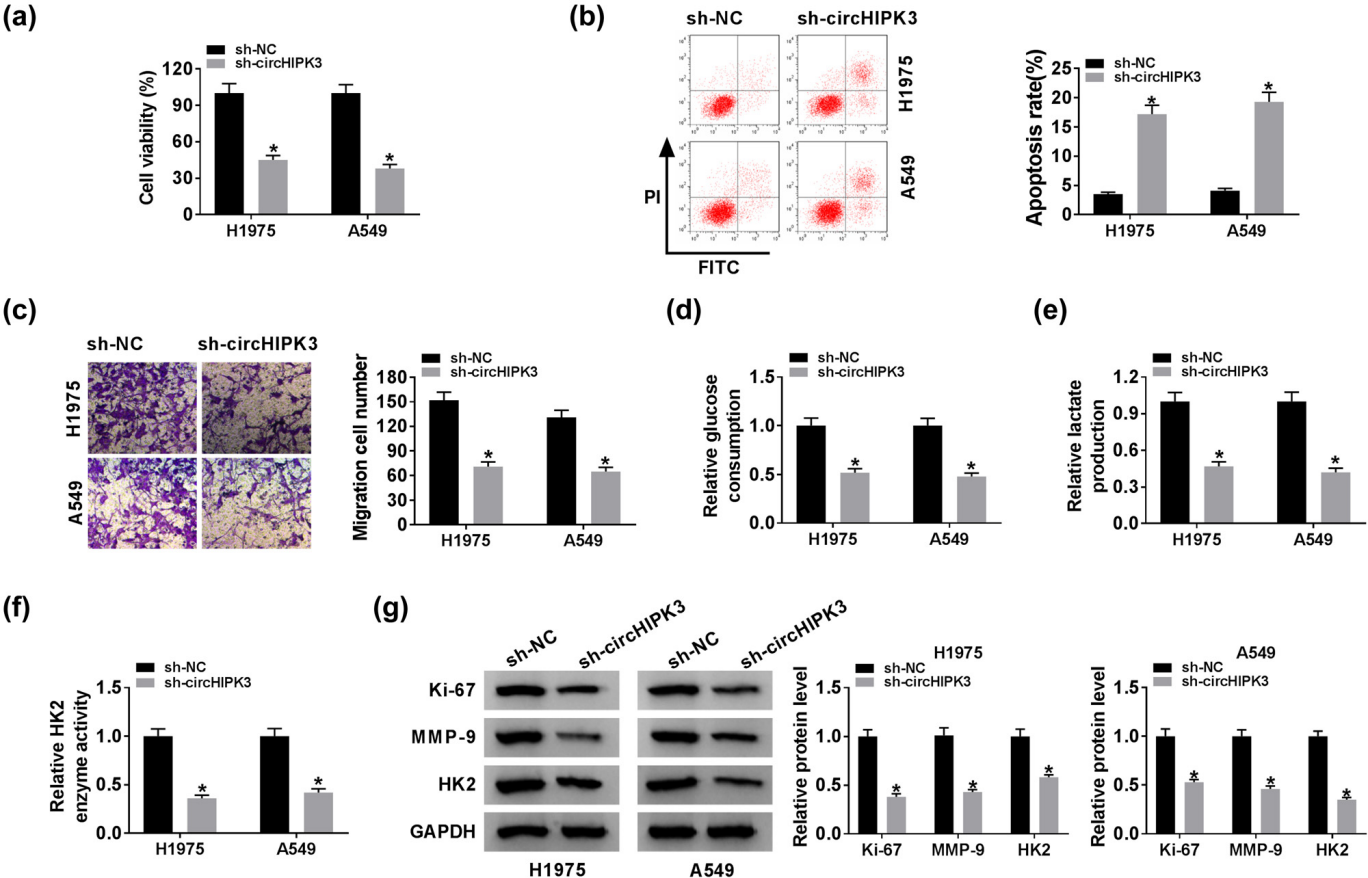
CircHIPK3 knockdown repressed cell proliferation, migration, and glycolysis while facilitated apoptosis in lung cancer cells. Lung cancer H1975 and A549 cells were introduced with sh-circHIPK3 or sh-NC. (a) CCK-8 assay for the cell viability of the treated H1975 and A549 cells. (b) Flow cytometry for the apoptosis of the transfected H1975 and A549 cells. (c) Transwell assay for the migration ability of the treated lung cancer cells. (d–f) Assessment of the glucose consumption, lactate production, and the enzyme activity of HK2. (g) Western blot analysis for the protein levels of Ki-67, MMP-9, and HK2 in transfected cells. *P < 0.05.

**Figure A1:**
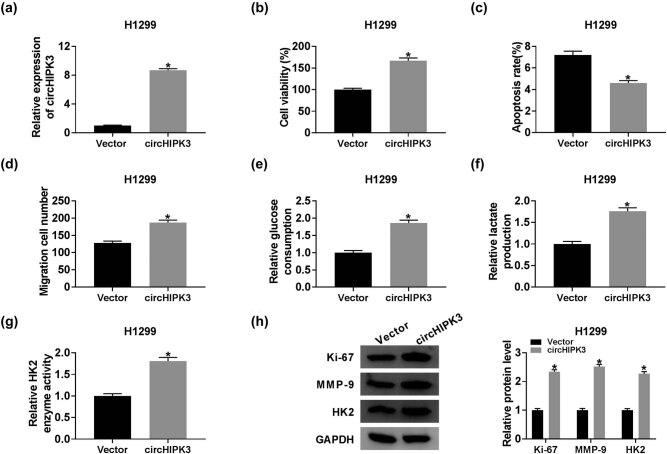
Upregulation of circHIPK3 facilitated cell proliferation, migration, and glycolysis but repressed apoptosis in lung cancer cells. H1299 cells were transfected with Vector or circHIPK3. (a) QRT-PCR assay for the relative expression of circHIPK3 in transfected cells. (b) CCK-8 assay for the cell viability of the transfected cells. (c) Flow cytometry for the apoptosis of the transfected cells. (d) Transwell assay for the migration capacity of the transfected cells. (e–g) Assessment of glucose consumption, lactate production, and the enzyme activity of HK2 in transfected cells. (h) Western blot assay for the protein expression of Ki-67, MMP-9, and HK2 in transfected cells. *P < 0.05.

Then, a series of loss-of-function experiments were performed in H1975 and A549 cells with circHIPK3 knockdown. CCK-8 assay witnessed the decline in the cell viability of H1975 and A549 cells with circHIPK3 knockdown (Figure 2a). As shown in Figure 2b, downregulation of circHIPK3 elevated the apoptosis rate of lung cancer cells, when compared with the cells transfected with sh-NC. The data from the transwell assay implied that circHIPK3 deficiency reduced the migration ability of lung cancer cells (Figure 2c). HK2 is an essential enzyme during the glycolysis process [23]. Using commercial kits, we found that circHIPK3 knockdown reduced the glucose consumption, lactate production, and the enzyme activity of HK2, suggesting that circHIPK3 knockdown weakened glycolysis in lung cancer H1975 and A549 cells (Figure 2d–f). Western blot analysis manifested that the silencing of circHIPK3 downregulated the protein levels of Ki-67, MMP-9, and HK2 in H1975 and A549 cells (Figure 2g). On the contrary, we also manifested that the upregulation of circHIPK3 facilitated cell proliferation, migration, and glycolysis but repressed apoptosis in H1299 cells (Figure A1). Taken together, silencing of circHIPK3 inhibited cell proliferation, migration, and glycolysis, while promoted apoptosis in lung cancer cells.

### 3.3 MiR-381-3p served as a target of circHIPK3

**Figure 3:**
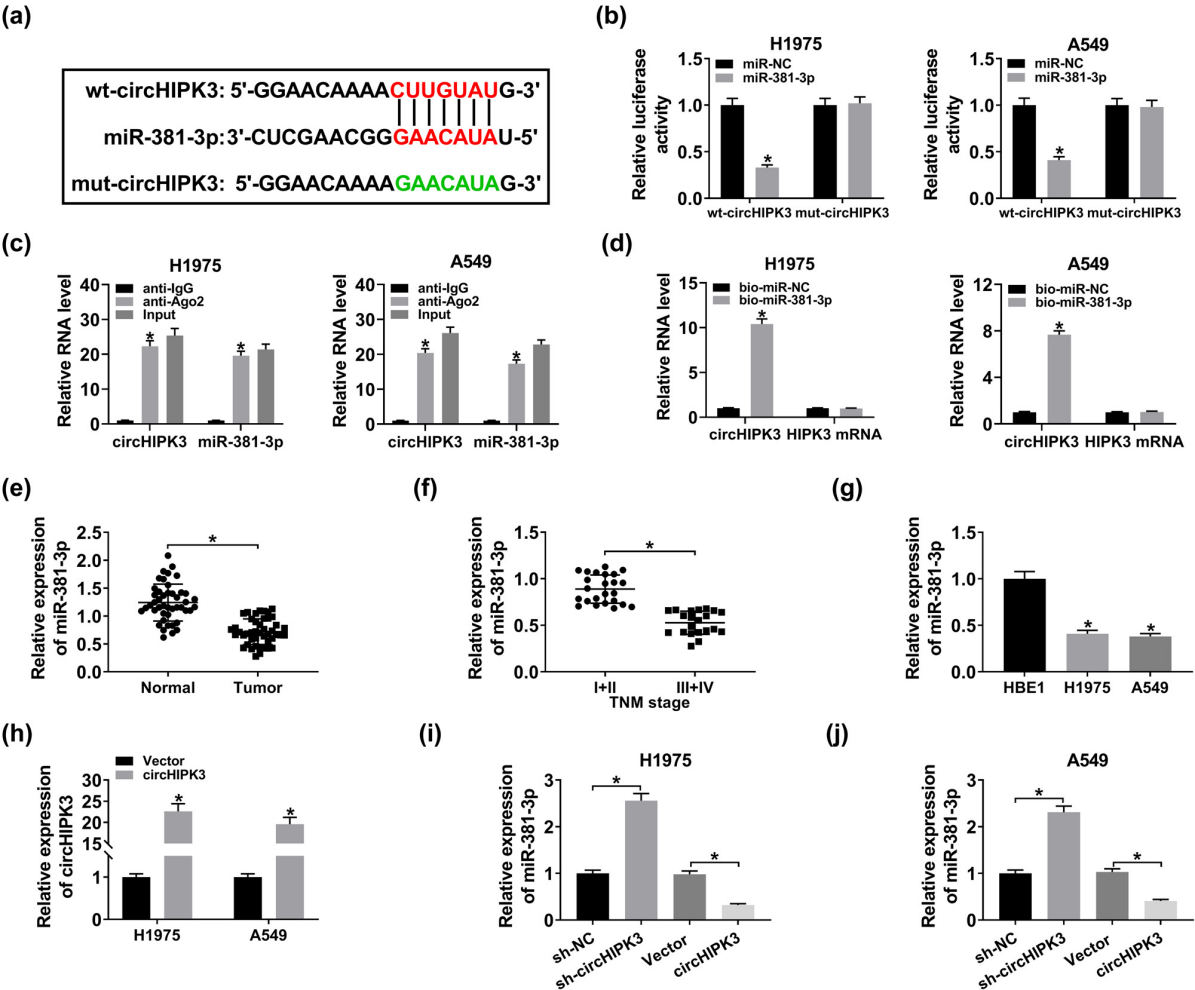
MiR-381-3p served as a target of circHIPK3. (a) Partial sequence of circHIPK3 and the binding sites for miR-381-3p. (b) Dual-luciferase reporter assay for the luciferase activity of wt-circHIPK3 and mut-circHIPK3 in cotransfected H1975 and A549 cells. (c) RIP and qRT-PCR assays for the binding efficiency of circHIPK3 and miR-381-3p in H1975 and A549 cells. (d) Pull-down assay for the binding ability between circHIPK3 and miR-381-3p in H1975 and A549 cells. (e–g) QRT-PCR assay for the relative expression of miR-381-3p in lung cancer tissues and normal tissues (e), in lung cancer tissues at stages I and II and at stages III and IV (f), as well as in HBE1 cells and lung cancer H1975 and A549 cells (g). (h) QRT-PCR assay for the relative expression of circHIPK3 in H1975 and A549 cells transfected with Vector or circHIPK3. (i and j) QRT-PCR assay for the relative expression of miR-381-3p in H1975 and A549 cells transfected with sh-NC, sh-circHIPK3, Vector, or circHIPK3. *P < 0.05.

Based on the forecast of starBase v3.0, we found that circHIPK3 (chr11: 33308803–33308809) could bind to miR-381-3p (Figure 3a). For the binding assay of circHIPK3 and miR-381-3p, we performed a dual-luciferase reporter assay and discovered that the introduction of miR-381-3p apparently lowered the luciferase activity of wt-circHIPK3 in both H1975 and A549 cells, while no significant change in the luciferase activity of mut-circHIPK3 was observed (Figure 3b). RIP assay was performed to investigate the binding efficiency of circHIPK3 and miR-381-3p. The results showed that circHIPK3 and miR-381-3p were highly enriched by anti-Ago2 antibody relative to anti-IgG antibody, indicating that circHIPK3 and miR-381-3p were present in RNA-induced silencing complex (RISC) (Figure 3c). Data from the pull-down assay showed that the bio-miR-381-3p-enriched circHIPK3 content was increased in contrast to the bio-miR-NC-enriched one. Besides, the level of HIPK3 mRNA changed little (Figure 3d). In sum, circHIPK3 and miR-381-3p could combine with each other in H1975 and A549 cells. QRT-PCR results revealed that the miR-381-3p expression was markedly reduced in lung cancer tissues with respect to adjacent normal tissues and was also downregulated in lung cancer tissues at stages III and IV when compared with tissues at stages I and II (Figure 3e and f). As expected, miR-381-3p expression in H1975 and A549 cells was lower than that in HBE1 cells (Figure 3g). To estimate the effects of circHIPK3 on the expression of miR-381-3p, H1975, and A549 cells with circHIPK3 upregulation were successfully constructed (Figure 3h). Then, qRT-PCR showed that the miR-381-3p level was increased in H1975 and A549 cells with circHIPK3 knockdown but reduced in cells with circHIPK3 overexpression (Figure 3i and j). To sum up, circHIPK3 targeted miR-381-3p and negatively regulated miR-381-3p expression in lung cancer cells.

### 3.4 MiR-381-3p interference alleviated circHIPK3 knockdown-induced damage on lung cancer progression

**Figure 4:**
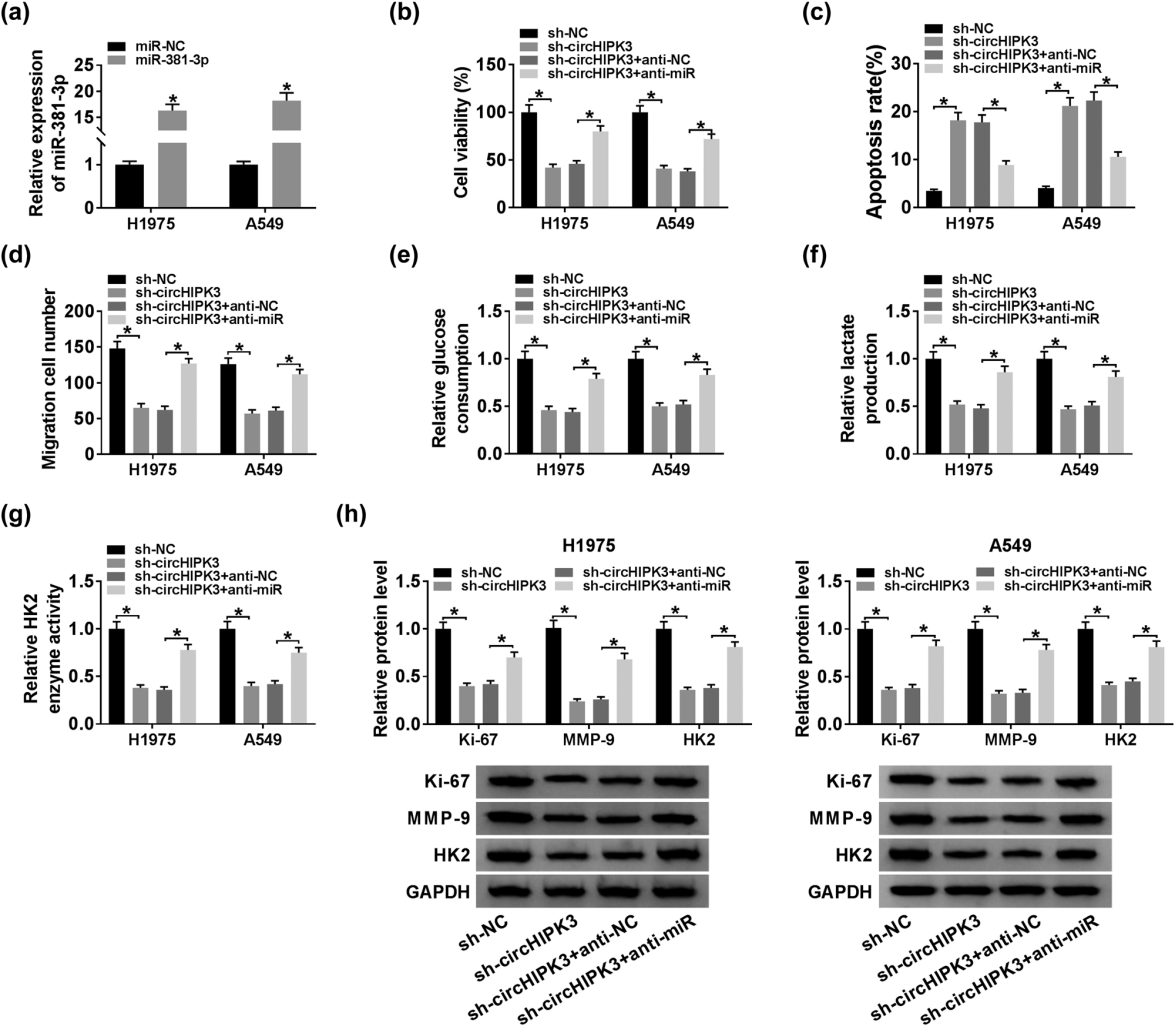
MiR-381-3p interference alleviated circHIPK3 knockdown-induced damage on lung cancer progression. (a) QRT-PCR assay for the relative expression of miR-381-3p in H1975 and A549 cells transfected with miR-NC or miR-381-3p. (b–h) H1975 and A549 cells were transfected with sh-NC, sh-circHIPK3, sh-circHIPK3 + anti-NC, or sh-circHIPK3 + anti-miR. (b) CCK-8 assay for the cell viability of the transfected H1975 and A549 cells. (c) Flow cytometry for the apoptosis of the H1975 and A549 cells. (d) Transwell assay for the migration capacity of the H1975 and A549 cells. (e–g) Assessment of the glucose consumption, lactate production, and the enzyme activity of HK2 of the H1975 and A549 cells. (h) Western blot assay for the protein expression of Ki-67, MMP-9, and HK2 in transfected cells. *P < 0.05.

As shown in Figure 4a, H1975 and A549 cells with miR-381-3p upregulation were successfully constructed. As miR-381-3p was a target of circHIPK3, we guessed that circHIPK3 exerted its function in lung cancer progression by sponging miR-381-3p. To validate our inference, a series of rescue experiments were implemented. H1975 and A549 cells were transfected with sh-NC, sh-circHIPK3, sh-circHIPK3 + anti-NC, or sh-circHIPK3 + anti-miR. Loss of miR-381-3p relieved the declined cell viability and migration in H1975 and A549 cells caused by circHIPK3 knockdown (Figure 4b and d). Flow cytometry suggested that circHIPK3 knockdown-induced apoptosis in the two cell lines was weakened by the introduction of anti-miR (Figure 4c). Moreover, circHIPK3 knockdown-induced glycolysis repression was also attenuated by miR-381-3p inhibitor, which was evidenced by the increase in the glucose consumption, lactate production, and enzyme activity of HK2 in cells co-transfected with sh-circHIPK3 and anti-miR (Figure 4e–g). Besides, the downregulation of the protein expression of Ki-67, MMP-9, and HK2 triggered by circHIPK3 deficiency was attenuated by the co-transfection with anti-miR (Figure 4h). The above data implied that circHIPK3 knockdown weakened lung cancer progression, at least in part, through upregulating miR-381-3p.

### 3.5 CircHIPK3 knockdown blocked the AKT/mTOR pathway via targeting miR-381-3p

**Figure 5:**
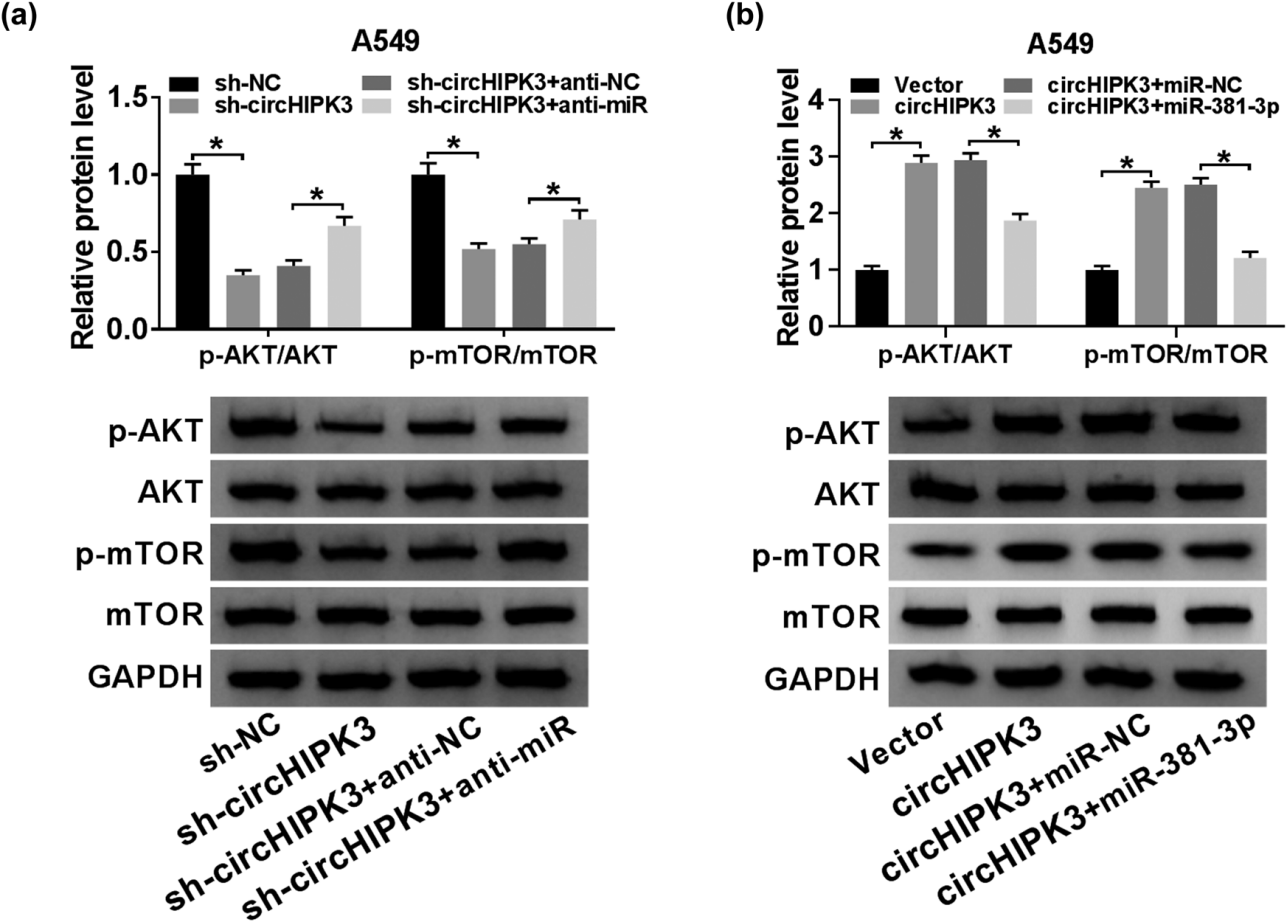
CircHIPK3 knockdown blocked the AKT/mTOR pathway via targeting miR-381-3p. (a) Western blot assay for the levels of p-AKT, AKT, p-mTOR, and mTOR in A549 cells transfected with sh-NC, sh-circHIPK3, sh-circHIPK3 + anti-NC, or sh-circHIPK3 + anti-miR. (b) Western blot assay for the levels of p-AKT, AKT, p-mTOR, and mTOR in A549 cells transfected with Vector, circHIPK3, circHIPK3 + miR-NC, or circHIPK3 + miR-381-3p. *P < 0.05.

AKT/mTOR pathway has been confirmed to be associated with cell growth, metabolism, and survival [24]. Here, we investigated the impact of circHIPK3 on the AKT/mTOR pathway in A549 cells. Western blot assay was employed to analyze the protein levels of AKT and mTOR, as well as their phosphorylation status. As shown in Figure 5a, A549 cells transfected with sh-circHIPK3 exhibited decreased levels of p-AKT/AKT and p-mTOR/mTOR versus cells transfected with sh-NC, which was reversed by addition of anti-miR. Similarly, elevated p-AKT/AKT and p-mTOR/mTOR levels were observed in A549 cells transfected with circHIPK3 with respect to cells transfected with Vector, while co-transfection with miR-381-3p abrogated the upregulation (Figure 5b). In conclusion, circHIPK3 could activate the AKT/mTOR pathway via sponging miR-381-3p.

### 3.6 CircHIPK3 knockdown suppressed the tumor growth by sponging miR-381-3p through the AKT/mTOR pathway

**Figure 6:**
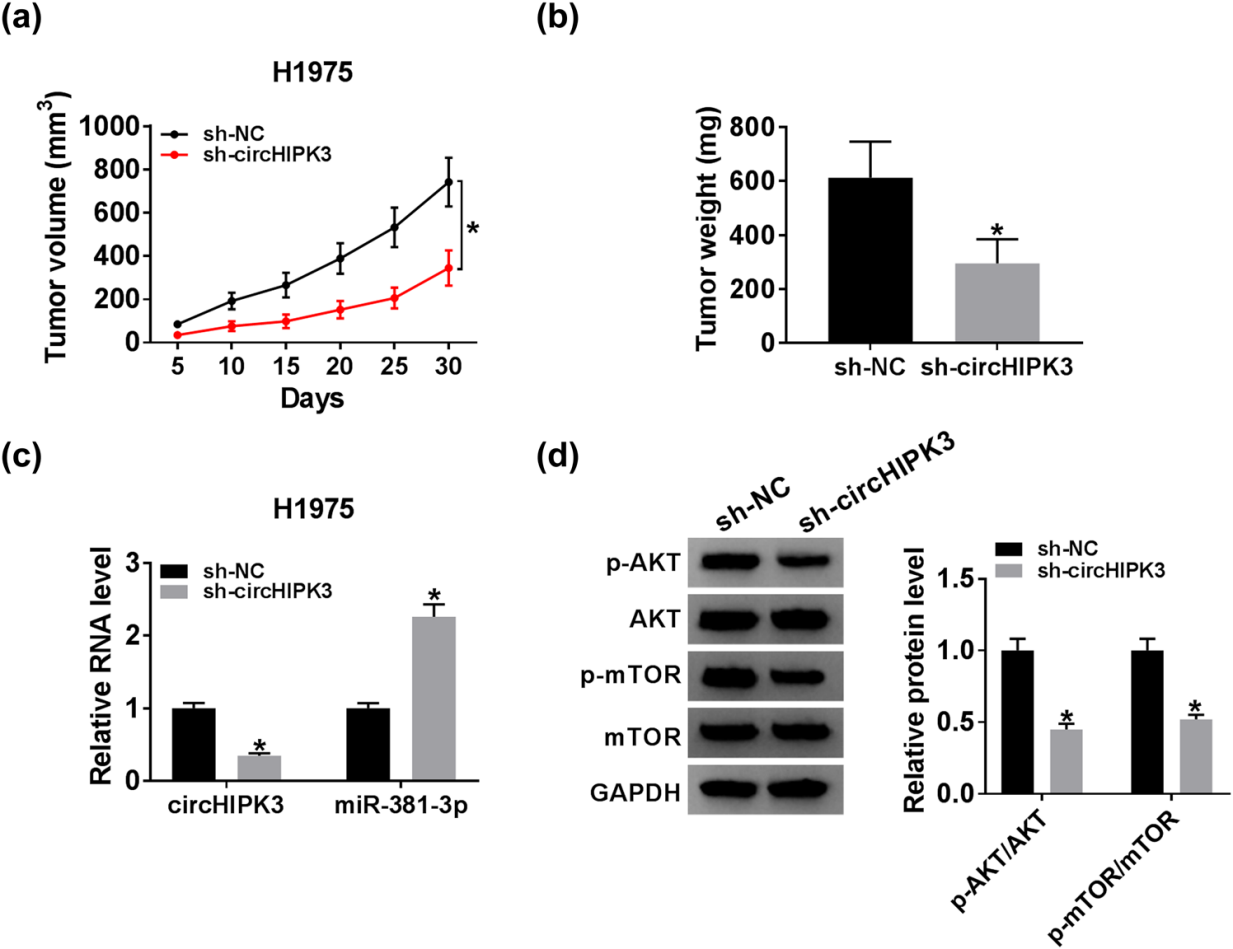
CircHIPK3 knockdown suppressed tumor growth by sponging miR-381-3p through AKT/mTOR pathway. Nude mice were subcutaneously implanted with H1975 cells stably transfected with sh-circHIPK3 or sh-NC. (a) The volume of generated tumors. (b) The weight of formed tumors. (c) QRT-PCR assay for the relative expression of circHIPK3 and miR-381-3p in generated tumors. (d) Western blot assay for the levels of p-AKT, AKT, p-mTOR, and mTOR in formed tumors. *P < 0.05.

Nude mice xenograft tumor experiments were implemented to clarify the role of circHIPK3 *in vivo*. As illustrated in Figure 6a and b, the volume and weight of formed tumors from nude mice in the sh-circHIPK3 group were reduced when compared with those in the sh-NC group, indicating that the loss of circHIPK3 repressed the tumor growth. In addition, qRT-PCR revealed that circHIPK3 expression was declined, while miR-381-3p expression was increased in the sh-circHIPK3 group (Figure 6c). We also found that the levels of p-AKT/AKT and p-mTOR/mTOR were decreased in the sh-circHIPK3 group relative to the sh-NC group (Figure 6d). Altogether, circHIPK3 knockdown inhibited lung cancer progression, at least in part, by targeting miR-381-3p to regulate the AKT/mTOR pathway *in vivo*.

## 4 Discussion

Lung cancer remains the most lethal cancer all over the world, because a large portion of patients are diagnosed at advanced stages [1]. In the current study, we identified an oncogenic circRNA circHIPK3 in this malignancy. Based on our experimental data, we concluded that circHIPK3 promoted lung cancer progression, at least in part, via sponging miR-381-3p and modulating the AKT/mTOR signaling pathway.

Dysregulation of circHIPK3 has been detected in certain human diseases, including upregulation in prostate cancer, NPC, glioma, and lung cancer [9,10,11,12] as well as downregulation in preeclampsia [25]. Here, we also detected the upregulation of circHIPK3 in lung cancer tissues and cells with respect to the corresponding controls. Functional assays in H1975 and A549 cells as well as in nude mice xenograft model were performed to explore the role of circHIPK3 in lung cancer. Our data showed that depletion of circHIPK3 resulted in the striking inhibition of cell proliferation, migration, glycolysis, and tumor growth, as well as the obvious promotion of apoptosis in lung cancer cells.

Previous literature suggested that circHIPK3 improved NSCLC progression through inducing the upregulation of miR-149-mediated FOXM1, resulting in the proliferation, metastasis, and tumorigenesis promotion of NSCLC cells [26]. Another report indicated that circHIPK3 played an oncogenic role in lung cancer and acted as a pivotal autophagy controller via miR-124-3p/STAT3/PRKAA axis, and its potential of being prognosis biomarker and treatment target for lung cancer was confirmed as well [12]. According to the ceRNA hypothesis, we used starBase v3.0 to search the target miRNA of circHIPK3 to better understand its mechanism of action in lung cancer. We found that miR-381-3p (GAACAUA) could bind to circHIPK3 (CUUGUAU). Moreover, miR-381-3p expression was lower in lung cancer tissues and H1975 and A549 cells than that in the corresponding controls. We also detected the circHIPK3-induced negative regulation on miR-381-3p expression. Functionally, circRNAs could sponge miRNAs, acting as ceRNAs, to reduce the activity of miRNAs and inhibition of their target mRNAs, perhaps inducing miRNA degradation by some means [27]. The tumor suppressor role of miR-381-3p has been demonstrated, including inhibiting cell proliferation, metastasis, and facilitating cell cycle arrest and apoptosis [16,18]. In addition, miR-381-3p had a connection with inflammatory responses in mouse models of spinal cord injury and acute respiratory distress syndrome [28,29]. In NSCLC, miR-381-3p was sponged by circFGFR1, an oncogenic circRNA functioning in proliferation, metastasis, and immune evasion capacities of tumor cells [19]. Hence, miR-381-3p was involved in many diseases with diverse functions. In this project, we first validated that miR-381-3p was targeted by circHIPK3 through dual-luciferase reporter, RIP, and pull-down assays. Furthermore, we also substantiated that miR-381-3p inhibitor reversed circHIPK3 knockdown-mediated reduced development of lung cancer, signifying that circHIPK3 played pro-tumor role in lung cancer, at least in part, by downregulating miR-381-3p.

PI3K/AKT/mTOR signaling pathway is implicated with various cellular behaviors of lung cancer cells, such as cell growth, metastasis, differentiation, survival, adhesion, and resistance to clinical treatment. Specific inhibitors of this transduction pathway could be used for NSCLC therapy in the clinic [30,31]. In this work, circHIPK3 activated the AKT/mTOR signaling pathway in H1975 and A549 cells by sponging miR-381-3p. We also demonstrated that the lack of circHIPK3 might block tumorigenesis of lung cancer H1975 cells by damaging the activation of AKT/mTOR signaling pathway.

There are still some limitations to this project. CeRNA network circRNA/miRNA/mRNA in lung cancer was widely identified [6,7,32,33]. Therefore, the downstream mRNA of the circHIPK3/miR-381-3p axis is worthy of further exploration to enrich our cognition on the mechanism of circHIPK3 in lung cancer.

In conclusion, this work elucidated that depletion of circHIPK3 repressed proliferation, migration, and glycolysis and contributed to apoptosis of lung cancer cells *in vitro*, as well as blocked tumor growth *in vivo*, at least in part, by absorbing miR-381-3p and modulating the AKT/mTOR signaling pathway, affording a potential therapeutic molecular target for lung cancer.
